# Synthesis and Characterization of an Alumina Forming Nanolaminated Boride: MoAlB

**DOI:** 10.1038/srep26475

**Published:** 2016-05-25

**Authors:** Sankalp Kota, Eugenio Zapata-Solvas, Alexander Ly, Jun Lu, Omar Elkassabany, Amanda Huon, William E. Lee, Lars Hultman, Steve J. May, Michel W. Barsoum

**Affiliations:** 1 Drexel University, Department of Materials Science & Engineering, Philadelphia, Pennsylvania 19104, United States; 2 Centre for Nuclear Engineering, Department of Materials, Imperial College London, London, SW7 2AZ, United Kingdom; 3 Drexel University, Department of Mechanical Engineering and Mechanics, Philadelphia, 19104, United States; 4 Linköping University, Department of Physics, Chemistry and Biology (IFM), Linköping, SE-58183, Sweden

## Abstract

The ‘MAlB’ phases are nanolaminated, ternary transition metal borides that consist of a transition metal boride sublattice interleaved by monolayers or bilayers of pure aluminum. However, their synthesis and properties remain largely unexplored. Herein, we synthesized dense, predominantly single-phase samples of one such compound, MoAlB, using a reactive hot pressing method. High-resolution scanning transmission electron microscopy confirmed the presence of two Al layers in between a Mo-B sublattice. Unique among the transition metal borides, MoAlB forms a dense, mostly amorphous, alumina scale when heated in air. Like other alumina formers, the oxidation kinetics follow a cubic time-dependence. At room temperature, its resistivity is low (0.36–0.49 μΩm) and – like a metal – drops linearly with decreasing temperatures. It is also a good thermal conductor (35 Wm^−1^K^−1^ at 26 °C). In the 25–1300 °C temperature range, its thermal expansion coefficient is 9.5 × 10^−6 ^K^−1^. Preliminary results suggest the compound is stable to at least 1400 °C in inert atmospheres. Moderately low Vickers hardness values of 10.6 ± 0.3 GPa, compared to other transition metal borides, and ultimate compressive strengths up to 1940 ± 103 MPa were measured at room temperature. These results are encouraging and warrant further study of this compound for potential use at high temperatures.

Binary transition metal borides and carbides are among the hardest and most refractory materials known. In addition, many of them have a unique combination of mechanical, electronic, and thermal properties that make them technologically important for applications such as wear resistant coatings^1^, primary battery electrodes^2^,^3^, chemical catalysis^4^,^5^, and high-temperature structural materials^6^. However, their use, especially in bulk form, is often limited by high processing costs and, more importantly, poor oxidation resistance when heated in air.

In general, the aforementioned properties also apply to most ternary transition metal borides, carbides, and nitrides. Notable exceptions are the M_n+1_AX_n_ (MAX) phases, a family of layered early transition metal carbides and nitrides, where M is an early transition metal, A is a Group IIIA-IVA element, and X is C and/or N, and n = 1, 2 or 3 (Space Group *P6*_*3*_*/mmc*)^7^. The crystal structure of the MAX phases comprises a ‘M_n+1_X_n_’ sublattice interleaved with monolayers of the ‘A’ element. This unique, layered structure results in properties that combine those of their MX binary carbide/nitride counterparts and transition metals making some MAX phases (e.g. Ti_2_AlC, Ti_3_SiC_2_) thermal shock resistant, readily machinable, resistant to high-temperature oxidation, mechanically rigid and plastic at high temperatures^8^,^9^,^10^.

Despite the extensive compositional variability possible for the MAX phases, phases where X = B do not exist. However, the M_2_AlB_2_-type (space group *Cmmm*) and MAlB-type (space group *Cmcm*) ternary transition metal borides – discovered by Jeitschko^11^,^12^–are close structural analogs to the MAX phases in that a transition metal boride sublattice is interleaved by one or two Al layers, respectively. To date, studies on these ternary borides have focused mostly on single-crystal growth and on determining their crystal structures. For example, Ade *et al.* recently synthesized single crystals of several previously reported M_2_AlB_2_ (M = Cr, Mn, Fe) and MAlB (M = Mo, W) compounds, and discussed their structural relationship to other transition metal borides and their similarity to the MAX phases^13^. Isostructural compounds with M-site solid solutions, viz. (Mo_x_,Me_1−x_)AlB, where Me = Cr, W^14^ and (Fe_2_,Me_2−x_)AlB_2_, where Me = Cr, Mn, have been also recently synthesized^15^.

A few studies investigated the electronic and magnetocaloric properties of polycrystalline (Fe_2_,Me_2−x_)AlB_2_ samples^16^,^17^. Okada *et al.* reported that single crystals of (Mo_x_,Cr_1−x_)AlB and (Mo_x_,W_1−x_)AlB have low electrical resistivities that vary between 0.65 to 2.45 μΩm and Vickers hardness (H_V_) values ranging from 10 to 20 GPa. Differential thermal analysis (DTA) on powders revealed that MoAlB and WAlB powders are only stable in air up to 800 °C. Among these, MoAlB is particularly attractive to study in bulk form because of its known thermodynamic stability^18^, relatively lower hardness (H_V_ = 10.3 GPa) compared to WAlB (H_V_ = 19.3 GPa), and higher electrical conductivity than WAlB.

As noted above, one of the major disadvantages of all transition metal borides, both binary and ternary, known to date, is their propensity for oxidation when heated in air at high temperatures^19^. Given the structural similarities between the ternary compound MoAlB and the MAX phases, and the relatively high Al content in the former, it was postulated that, like Ti_2_AlC and Cr_2_AlC^20^,^21^, a protective alumina layer would form upon heating in air. As shown herein, our postulate was correct and heating polycrystalline samples of MoAlB in air, to temperatures as high as 1400 °C, resulted in formation of a passivating, mostly amorphous, alumina layer. The purpose of this paper is to report on the synthesis of MoAlB using a reactive hot pressing technique. In addition to studying its oxidation resistance, we also report on some of its electrical, thermal, and mechanical properties.

## Results and Discussion

### Synthesis

**Figure 1:**
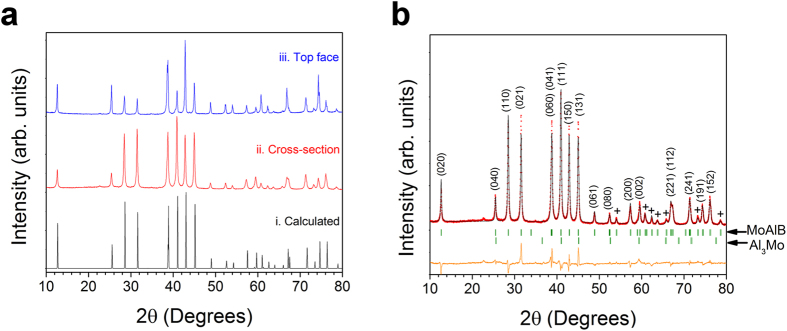
(**a**) XRD diffractograms of (i) calculated 2θ positions, (ii) hot-pressed cross section, and, (iii) top surface of the hot-pressed sample; (**b**) Rietveld refinement of the HP4 cross-section’s diffractogram with the observed pattern (red), calculated pattern (black), and difference in observed and calculated intensities (orange). Green dashes show calculated 2θ positions for MoAlB (top row) and Al_3_Mo (bottom row). Diffraction peaks marked with (+) also belong to MoAlB.

X-ray diffractograms of the hot-pressed sample’s polished cross-section (HP4) and polished top surface are in good agreement with the calculated diffractogram reported by Ade *et al.*^13^, as shown in Fig. 1a. However, the experimentally observed peak intensity ratios of the hot-pressed sample differed from those of the calculated diffractogram. The relatively higher intensities of the {*0k0*} peaks of the top surface compared to those of the cross-section indicate that hot pressing helped to preferentially orient the [010] axis of the grains parallel with the hot pressing direction. XRD also showed the presence of Al_3_Mo and an unidentified impurity with minor peaks at 22.8^o^ and 43.9^o^. In contrast to HP4, the HP2 sample (see Methods) had more porosity and Al_2_O_3_ impurities, but negligible amounts of intermetallic impurities (Fig. S1a).

Rietveld refinement on the polished HP4 cross-section’s diffractogram showed the sample to be predominantly single phase MoAlB with impurities of 3 vol.% Al_3_Mo (Fig. 1b). Lattice constants of a = 3.21 Å, b = 13.98 Å, and c = 3.10 Å were obtained from Rietveld refinement and compared well those reported for MoAlB by Ade *et al.* and Okada^13^,^22^. A χ^2^ value of 8.9 was obtained for the refinement despite accounting for all major peaks. This high χ^2^ value is presumably due to a preferred orientation relationship too complex to be determined by 1-dimensional XRD used herein. Indirect evidence for this conjecture is the fact that Rietveld refinement for MoAlB powders, synthesized in the tube furnace, showed a much better fit (χ^2^ = 3.9). In this case, the powder was predominantly single phase, with Al_2_O_3_ (8 vol.%) and unreacted Al (2 vol.%) as impurities (Fig. S2). Not surprisingly, in contrast to the hot-pressed sample, **t**he experimentally observed peak intensity ratios were quite similar to the calculated diffractogram (Fig. S2). When the refined lattice constants of the sample HP4 and the powders are compared with previous results (Table S1), excellent agreement is found.

**Figure 2:**
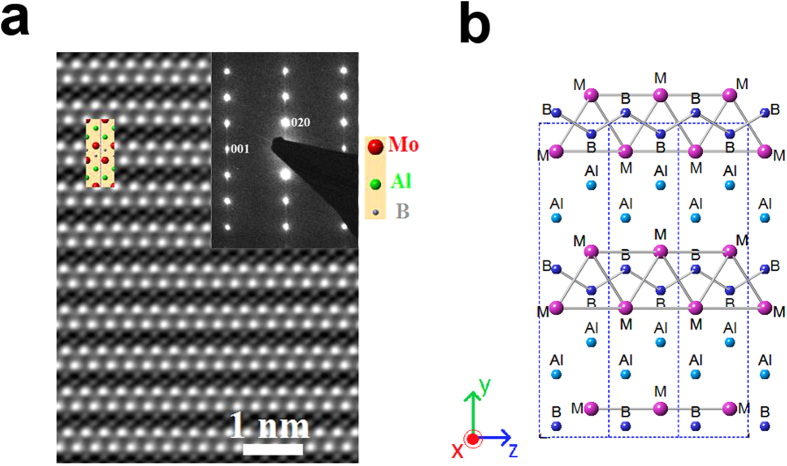
(**a**) HRSTEM image of MoAlB along the [100] zone axis. Insets shows SAED pattern along the [100] zone axis (top right) and the positions of Mo, Al, and B atoms (top left); (**b**) Crystal structure of MoAlB viewed on the (100) plane.

In Fig. 2a, high-resolution scanning transmission electron microscopy (HRSTEM) along the [100] zone axis further confirms that the atomic layering - shown in Fig. 2b **-** is correct. Selected area electron diffraction (SAED) along the [100] zone axis (inset of Fig. 2a) confirms the orthorhombic symmetry of MoAlB. Interestingly, some grains contain stacking faults in which only one Al layer, instead of two, is sandwiched by the Mo-B layers (Fig. S3). However, the density of such stacking faults is low as judged by the weak diffraction streaks along [010] in the SAED pattern.

**Figure 3:**
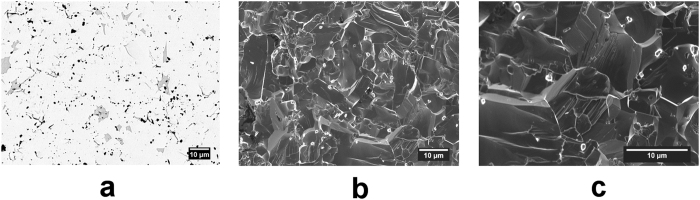
(**a**) Backscattered electron micrograph of the hot-pressed HP4 cross-section; (**b**) secondary electron micrograph of the fracture surface at low magnification; (**c**) fracture surface at higher magnification reveals striations and layered structure of grains.

A typical backscattered electron micrograph of the polished HP4 cross-section is shown in (Fig. 3a) where the presence of, at least three phases - a majority phase, and at least two minority phases – are evidenced. The areas of lightest contrast are the majority phase, MoAlB. In agreement with XRD, EDS shows that the impurity phase with intermediate contrast is an Al-Mo impurity with Al:Mo molar ratio of 2.5:1. Image analysis showed that 6 ± 2 vol.% of this impurity phase is present. The areas of darkest contrast, making about 3 ± 0.5 vol.% of the sample, are primarily Al_2_O_3_ impurities according to EDS. In contrast to HP4, the HP2 cross-section showed primarily Al_2_O_3_ impurities (up to 9 vol.%) and negligible amounts of intermetallic compounds, which is consistent with our XRD results (Fig. S1b).

The cross-sectional fracture surface of the HP4 sample (Fig. 3b) shows it to contain mostly elongated, plate-like grains. Upon fracture, the grains were cleaved exposing large facets, which are presumably the {*0k0*} planes. The same fracture surface, shown at higher magnification in Fig. 3c, reveals sheared areas and other striations characteristic of nanolaminated materials.

### Electronic Transport Properties

**Figure 4:**
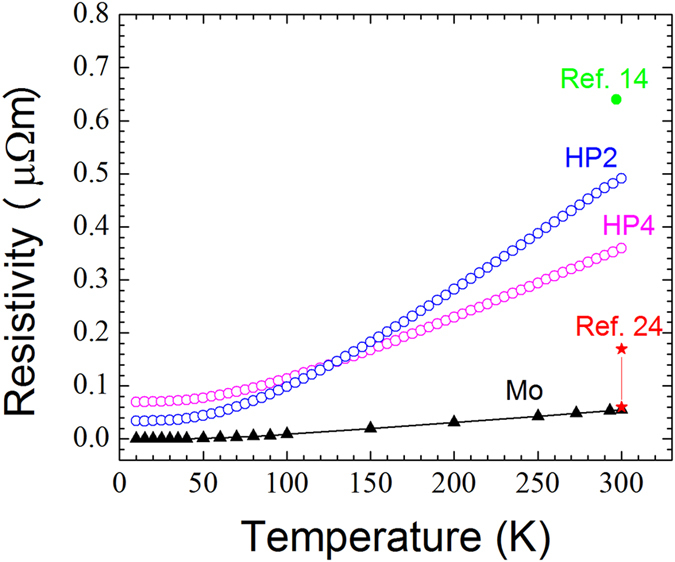
Resistivity vs. temperature of hot-pressed samples, MoAlB single crystals^14^,^24^, and pure Mo metal^23^.

The temperature dependence of the electrical resistivity (ρ) of the HP4 and HP2 samples is compared with that of pure Mo reported by Desai *et al.*^23^ in Fig. 4. At 300 K, the resistivity is 0.36 μΩm for HP4 and 0.49 μΩm for HP2. Like a metal, the resistivity of both samples increases linearly with temperature above 100 K. This temperature dependence can be fit to the following equation:


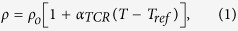


where T_ref_ is 300 K, T the absolute temperature, ρ_o_ is the resistivity at 300 K, and α_TCR_ is temperature coefficient of resistivity. Least-squares linear fitting in the 100–300 K temperature range results in a α_TCR_ = 0.0042 K^−1^ for HP2 and α_TCR_ = 0.0035 K^−1^ for HP4. A larger residual resistivity ratio, defined as ρ_300K_/ρ_10K_, for HP2 suggests that this sample is less defective. On the other hand, the larger resistivity above 140 K, and the larger α_TCR_ of HP2, are possibly due to its slightly lower density and the presence of more Al_2_O_3_ impurities than HP4. For comparison, the resistivity of pure Mo metal is 8.8 × 10^−6 ^μΩm and 0.055 μΩm at 10 K and 300 K, respectively.

At 300 K, Okada *et al.* reported a slightly higher value of resistivity (0.64 μΩm) when measured along the b-planes of MoAlB single crystals^14^. In contrast, the resistivity values reported by Sinel’nikova *et al.* (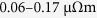) are much lower^24^. The reason for the large differences in reported literature values is unclear at this time. This comment notwithstanding, fabricating fully dense MoAlB samples with fewer impurities should further decrease the resistivity values at all temperatures.

### Thermal Properties

**Figure 5:**
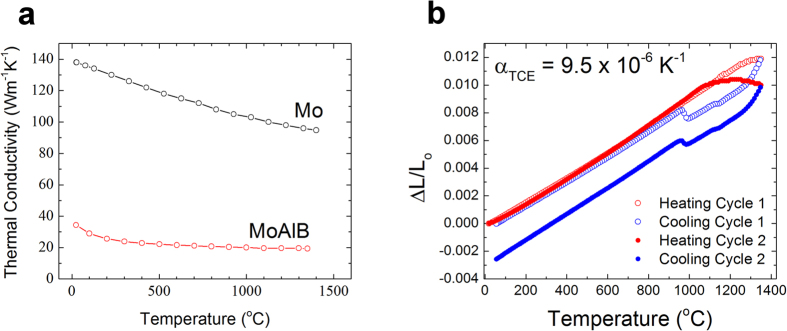
(**a**) Temperature dependence of thermal conductivity of hot-pressed MoAlB (HP4) and Mo metal^25^; (**b**) Temperature dependence of normalized thermal expansion measured by dilatometry in an Ar atmosphere. The first two cycles are shown.

The thermal conductivity (κ) of sample HP4 measured parallel with hot-pressing direction as a function of temperature from 25 to 1350 °C is shown in Fig. 5a. A constant heat capacity of 559.5 J kg^−1^K^−1^ was assumed as per the Neumann-Kopp rule to calculate the thermal conductivity. At 26 °C, the thermal conductivity is 35 Wm^−1^K^−1^ and steadily decreases to 19.4 Wm^−1^K^−1^ at 1350 °C. The thermal conductivity of Mo metal is shown in Fig. 5a for comparison and is roughly 5 times higher than that of MoAlB at all temperatures^25^.

Based on the results shown in Fig. 5b, the thermal expansion coefficient, CTE, of this compound was calculated to be 9.5 × 10^−6^ K^−1^ up to 1350 °C during heating. Upon cooling a non-linear contraction is observed from 1350 °C to 974 °C, before the expansion folds back onto the heating curve. When the same sample was heated a second time the same CTE was measured, but now the non-linear effect was magnified and a permanent shrinkage of the sample is observed. The origin of this anomaly is unclear at this time, and more work is ongoing to understand it. The value measured herein is higher than that of Mo metal (4.8 × 10^−6 ^K^−1^) or hot-pressed MoB (6.7 × 10^−6 ^K^−1^)^26^. The excellent adhesion of the protective alumina scales that form on MoAlB during high temperature oxidation (see below) can partially be explained by the closeness of its CTE at high temperatures to that of Al_2_O_3_, viz. 8.5 × 10^−6 ^K^−1^ 27.

Preliminary differential thermal analysis (DTA) and thermogravimetric analysis (TGA) on dense HP4 samples show no evidence of dissociation or melting up to 1400 °C. More work is ongoing to characterize the thermal stability of MoAlB at even higher temperatures.

### Mechanical Properties

**Figure 6:**
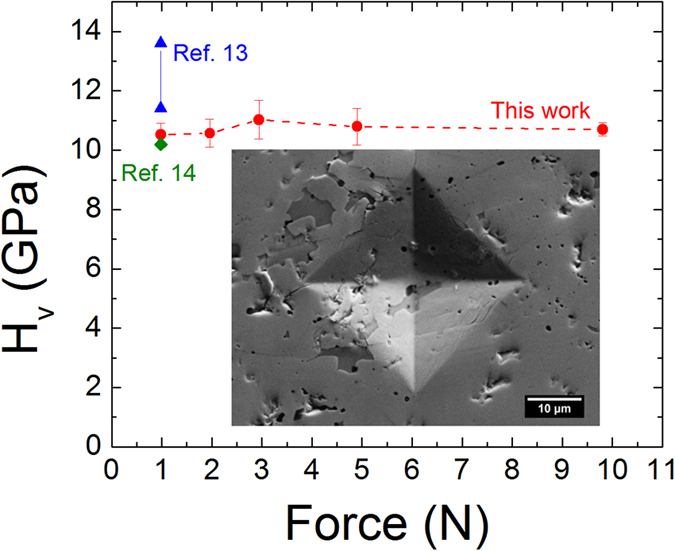
Vickers microindentation hardness as a function of indentation load. Inset shows indent formed under a 9.8 N load. Single crystal hardness values are shown for comparison^13^,^14^.

The Vickers hardness values (H_v_) measured at different loads on the cross-section are shown in Fig. 6. At all loads up to 9.8 N, the hardness was approximately constant at 10.6 ± 0.3 GPa, which agrees well with the hardness measured on the b-planes of MoAlB single crystals (10.3 ± 0.2 GPa) by Okada *et al.*^14^. In contrast, Ade *et al.* found the hardness on different crystal planes to range from 11.4–13.6 GPa^13^. At this time, we cannot comment on the effect of structural anisotropy on hardness, but it is clear that MoAlB is relatively soft compared to other borides such as MoB (H_v_ = 23 GPa)^19^, MoB_2_ (H_v_ = 21–27 GPa)^28^,^29^, and many other transition metal borides^19^. As the inset in Fig. 6 shows, no dominant cracks formed at the corners of the indents, even at the highest indentation load of 9.8 N, so MoAlB, like the MAX phases^9^, may be quite damage tolerant.

The test cylinders compressed perpendicular to the HP direction had an ultimate compressive strength, UCS, σ_+_=1940 ± 100 MPa; those compressed parallel with the HP direction had a UCS, σ_//_=1420 ± 300 MPa. Figure S4 shows the fracture surfaces after loading in the two perpendicular directions in relation the hot pressing direction. Two observations are salient. Firstly, the UCS values measured, especially the one close to 2 GPa, are quite high considering the size of the grains. Secondly, despite the fact that the most likely reason for the differences in strengths is the preferred orientation of the grains described above, these micrographs are not sufficiently different to make that case. More work is obviously needed. In both cases, brittle fracture occurred, and no yielding was observed prior to fracture. In general, cracks initiated at the base of the specimens and propagated upwards to create fracture surfaces often nearly parallel with the loading direction before the samples shattered into a few pieces.

### Oxidation Resistance

**Figure 7:**
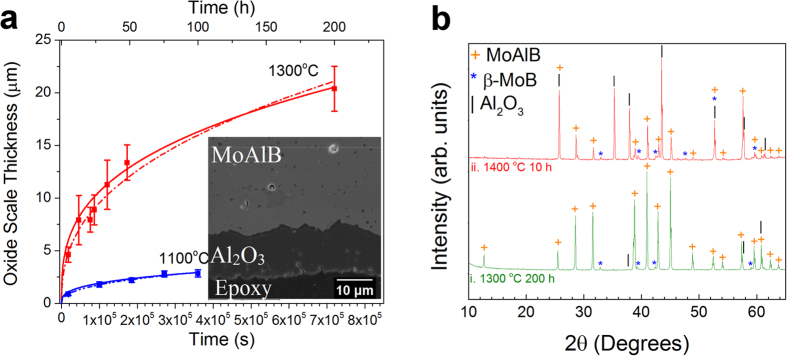
(**a**) Time dependence of the oxide scale thickness from isothermal oxidation testing at 1300 °C (red) and 1100 °C (blue). Solid curves show fits to a cubic law; dashed curves show fits to a power law (see text). Inset show MoAlB/oxide scale interface after 200 h at 1300 °C; (**b**) XRD after oxidation at (i) 1300 °C for 200 h and, (ii) 1400 °C for 10 h.

Oxidation at 1100 °C and 1300 °C resulted in the formation of dense, adherent oxide scales on the surface. A sample oxidized for 200 h at 1300 °C showed the presence of a 20 ± 2 μm thick scale, as shown in a cross-sectional micrograph (inset of Fig. 7a). Oxidation for 100 h at 1100 °C resulted in the formation of a 3 ± 0.4 μm thick scale. EDS near MoAlB/oxide interface (Fig. S5), formed after 200 h of oxidation at 1300 °C, revealed large differences in the relative atomic concentrations of Mo, Al, and O on the scale compared to the underlying MoAlB. It is clear that the nearly equal relative atomic concentrations of Mo and Al found at points 1, 4, and 5 correspond to pristine MoAlB. In contrast, the absence of Mo and an O:Al atomic ratio of 1.67 at points 6–10 suggest that Al preferentially diffused out of MoAlB and reacted with O to form an Al_2_O_3_ scale.

XRD of the samples oxidized for 1300 °C for 200 h (Fig. 7bi) shows faint diffraction peaks corresponding to the formation of β−ΜοΒ and Al_2_O_3_ (ICSD #01-071-1125). Clear XRD evidence for the formation of Al_2_O_3_, however, was only obtained when a sample was oxidized at 1400 °C for 10 h (Fig. 7bii). It follows that the alumina layers formed at temperatures as high as 1300 °C were mostly amorphous and quite resistant to crystallization. For example, when Ti_2_AlC, another alumina former, is oxidized, clear and sharp alumina peaks are observed in XRD diffraction patterns of samples oxidized in air at 1000 °C for 120 h^30^. This resistance to crystallization is quite unusual and warrants further work. Notably, when understood, it may be possible to synthesize an amorphous alumina that may flow like glass, an exciting prospect.

Since there was no strong evidence for other phases forming during oxidation, it is reasonable to assume the oxidation reaction is:


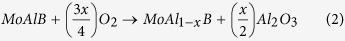


In other words, like in several alumina forming MAX phases^31^, one can conclude that MoAlB can exist with a deficiency of Al. Although the extent of that deficiency is unknown at the testing temperatures, phase equilibria of the Mo-Al-B system at 1000 °C support the idea that MoAlB can exist with a sub-stoichiometric content of Al to accommodate for the Al lost during oxidation^18^. From the fact that MoB does not precipitate below the alumina layer, it is possible that the B dissolves in, and diffuses out through the protective alumina layer. The same conclusion was reached for the fate of C during the oxidation of Ti_2_AlC^20^. However, more work needs to be done to clearly understand the role of Mo and B during high-temperature oxidation, and their role, if any, in preventing the crystallization of the alumina layer.

The oxidation kinetics were determined by measuring the scale thicknesses, x, as a function of time, t, at a fixed temperature. The results - shown in Fig. 7a – were fit to the well-known power law equation:


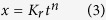


where K_r_ is the oxidation rate constant and n is the power law scale growth exponent. At 1300 °C, a good power law fit (R^2^ = 0.97) was obtained with n = 0.4. Similarly, at 1100 °C, a good power law fit (R^2^ = 0.99) was obtained with n = 0.4. Since these growth exponents are close to the value of n = 0.33 typical of alumina^32^, the data were also fit to the cubic rate law viz.


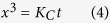


where K_c_ is the cubic rate constant. Figure 7a shows that very good fits were again observed at 1100 °C (R^2^ = 0.96) and 1300 °C (R^2^ = 0.99). Since cubic oxidation kinetics are rationalized on the basis of crystal growth, it is not clear why the kinetics observed here are cubic. TEM studies are ongoing to understand the exact oxidation mechanism.

Table 1 compares the K_c_ values obtained in this work with those of Ti_2_AlC and Ti_3_AlC_2_, which both form dense, crystalline alumina scales when heated in air^30^,^33^. For straightforward comparison of these materials, the weight gain rate constants of Ti_2_AlC and Ti_3_AlC_2_ were multiplied by 9.4 × 10^−10 ^m^9^kg^−3^ to obtain scale growth rate constants, which is based on the oxidation reaction that causes alumina to form on Ti_3_AlC_2_ and Ti_2_AlC^32^. MoAlB shows better oxidation resistance than Ti_2_AlC and Ti_3_AlC_2_ at 1100 °C. At 1300 °C, however, the oxidation resistances are comparable.

As noted above, similar thermal expansion coefficients for MoAlB (9.5 × 10^−6 ^K^−1^) and Al_2_O_3_ (8.5 × 10^−6 ^K^−1^) and high thermal conductivity of MoAlB suggest that this compound would be resistant to spallation of the protective oxide. Therefore, further investigations on the oxidation mechanisms and kinetics are warranted once the processing steps have been optimized.

It is interesting to compare the oxidation behavior of MoAlB with that of zirconium diboride (ZrB_2_), a leading candidate for high-temperature aerospace applications. Opeka *et al.* performed isothermal oxidation on nominally pure hot-pressed ZrB_2_ and demonstrated that active oxidation occurs above 1200 °C due to the evaporation of the B_2_O_3_ from the mixed ZrO_2_-B_2_O_3_ scale^34^, while other studies have demonstrated paralinear, or parabolic, oxidation kinetics for ZrB_2_ in the 1100–1400 °C range for short oxidation times (<5 h)^35^,^36^. In Opeka’s study, a 200 μm thick porous ZrO_2_ scale was observed after only 5 h at 1300 °C compared to the dense 5 μm thick Al_2_O_3_ scale that forms on MoAlB under the same conditions in the current study^34^.

### Summary

Reactive hot pressing of MoB and Al powders resulted in predominantly single phase, >94% dense MoAlB samples with ~9 vol.% secondary phases. HRSTEM images confirmed the atomically layered structure of MoAlB for the first time. Static oxidation testing for up to 200 h led to the formation of dense, adherent alumina scales that render MoAlB highly oxidation resistant up to at least 1300 °C.

MoAlB is a metallic conductor with a correspondingly high thermal conductivity. Also, its thermal expansion coefficient (9.5 × 10^−6 ^K^−1^) makes it compatible with many engineering alloys. The high compressive strength – comparable with that of alumina or silicon carbide - and relatively low hardness of MoAlB certainly warrant further investigation of the damage tolerance of this material at ambient and elevated temperatures. The properties of MoAlB measured herein are summarized in Table 2. Although these preliminary results are not optimized, MoAlB ceramics hold great promise as high temperature materials and coatings.

## Methods

### Processing

Dense, polycrystalline samples of MoAlB were synthesized by hot-pressing reactants together in a vacuum hot press (HP), as reported for several MAX phases^37^. First, molybdenum boride, MoB (>99%, <38 μm, Alfa Aesar, Ward Hill, MA, USA) and aluminum, Al (99.5%, <44 μm Alfa Aesar, Ward Hill, MA, USA), powders were ball milled in a molar ratio of 1.0 to 1.3 in a plastic container for 24 h. The mixture was loaded into a graphite foil lined cylindrical graphite die, heated to 1200 °C at a rate of 300 °C h^−1^ and pressed to a peak load corresponding to a stress of 39 MPa during the last 1 h of the temperature ramp. This temperature and pressure were held for 5.8 h (HP4 samples) or 5 h (HP2 samples), after which the hot press was allowed to cool. Unless otherwise stated, all characterization was performed on HP4 samples. The densities of the hot-pressed samples - as determined by Archimedes’ principle in water - were typically at least 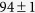
% of theoretical (6.45 g/cm^3^).

The same reactant mixture was cold-pressed to a load corresponding to 300 MPa and heated to 1000 °C for 15 h in a tube furnace with flowing argon, Ar, gas to obtain loosely sintered compacts. The latter were ground into powder with a drill bit and used for transmission electron microscopy (TEM) and high-resolution scanning transmission electron microscopy (HRSTEM) instead of the hot-pressed samples due to the difficulty of grinding down the latter.

### Characterization

X-ray diffraction (XRD) patterns of the hot-pressed samples and MoAlB powders were obtained on a powder diffractometer (SmartLab, Rigaku Corp., Tokyo, Japan) using Cu K_α_radiation. The FullProf Software suite was used to perform Rietveld refinement^38^,^39^. A scanning electron microscope, SEM (Zeiss Supra 50VP, Carl Zeiss SMT AG, Oberkochen, Germany), equipped with an energy-dispersive X-ray spectroscope (Oxford EDS, Oxfordshire, United Kingdom) was used to characterize the microstructure and the elemental compositions. The MoAlB powders were analyzed with TEM/SAED on a FEI Tecnai G2 TF20 UT (FEI, Hillsboro, Oregon, USA) equipped with a field emission gun operated at a voltage of 200 kV and HRSTEM by the Linköping double Cs corrected FEI Titan3 60–300 (FEI, Hillsboro, Oregon, USA) operated at 300 kV. The TEM specimen was prepared by first mixing the powder with glue, followed by heating, polishing down to 50 μm and then ion milling to make electron transparent.

The electrical resistivity was measured in the 10 K to 300 K temperature range using the 4-probe method in a physical property measurement system (Quantum Design, San Diego, CA, USA). Thin cross-sections (0.5–0.8 mm thick) of the HP2 and HP4 samples were cut with a high speed diamond saw, polished to a smooth finish with 800 grit SiC paper, and washed with ethanol prior to the measurements. The resistivity was measured by placing the electrodes on the cross-sections of these samples.

The thermal conductivity was measured parallel to the hot pressing direction using a laser flash instrument (Netzsch LFA 427, Selb, Upper Franconia, Germany) over the temperature range 25–1350 °C, at a heating rate of 10 °C/min on polished disks (10 mm diameter, 3 mm height) cut by electrical discharge machining (EDM). The coefficient of thermal expansion (CTE) was measured perpendicular to the hot pressing direction in a dilatometer in the 25 °C to 1350 °C temperature range on EDM’d cylinders (20 mm length, 6 mm diameter) under helium gas, using a dual-push-rod dilatometer (Netzsch DIL 402E, Selb, Upper Franconia, Germany). Thermogravimetric analysis (TGA) and differential thermal analysis (DTA) were performed simultaneously in Ar on hot-pressed samples from 25 °C to 1600 °C at a rate of 10 °C min.^−1^ (Netzsch STA 449F1, Selb, Upper Franconia, Germany).

The Vickers microindentation hardness was measured using a microindenter (LECO-M400 LECO Corp., St. Joseph, MI). Indentation loads between 0.98 and 9.8 N with a 15 s dwell time were used. Indent diagonals were measured using a scanning electron microscope, SEM (Zeiss Supra 50VP, Germany). The hardness values represent the average of at least three indents at each load.

The room temperature ultimate compressive strengths, UCSs, were measured using an Instron 5800R (Instron, Norwood, MA, USA) or MTS servo-controlled hydraulic system (MTS Systems Co., Eden Praire, MN, USA). Test cylinders (5 mm diameter, 13 mm long) were EDM’d lengthwise perpendicular to the HP direction and tested with no further preparation, as per ASTM C1424-10. Sample cylinders (5 mm diameter, 10 mm tall) were also EDM’d lengthwise parallel with the HP direction for comparison. The samples were compressed in displacement-control mode, at a rate of 0.3 mm/s until fracture.

The oxidation resistance was tested at 1100 °C, 1300 °C, and 1400 °C in static air for various times up to 200 h. The samples used were 4 × 4 × 4 mm^3^ cubes machined via EDM and polished with 1200 grit SiC polishing paper to a mirror-like finish. The oxidized samples were then mounted in epoxy, and again polished down to 1200 grit SiC paper in order to measure the thickness of the oxide scales in the SEM.

## Additional Information

**How to cite this article**: Kota, S. *et al.* Synthesis and Characterization of an Alumina Forming Nanolaminated Boride: MoAlB. *Sci. Rep.* **6**, 26475; doi: 10.1038/srep26475 (2016).

## Supplementary Material

Supplementary Information

## Figures and Tables

**Table 1:** 

	1100 °C	1300 °C	References
MoAlB	7.1 × 10^−23 ^m^3^/s	1.2 × 10^−20 ^m^3^/s	This work
Ti_2_AlC	1.0–1.8 × 10^−21 ^m^3^/s	1.4–4.8 × 10^−20 ^m^3^/s	30,33
Ti_3_AlC_2_	1.6 × 10^−21 ^m^3^/s	1.9 × 10^−20 ^m^3^/s	30,33

Comparison of the cubic oxidation rate constant K_c_ of MoAlB, Ti_2_AlC, and Ti_3_AlC_2_.

**Table 2:** 

Minimum relative density	94 ± 1%
Electrical Resistivity at 300 K, ρ_300_	0.35–0.49 μΩm
Temperature Coefficient of Resistivity, α_TCR_	0.0035–0.0042 K^−1^
Decomposition/Melting Temperature	>1400 °C
Vickers Microindentation Hardness, H_V_	10.7 + 0.3 GPa
Thermal Expansion Coefficient, CTE	9.5 × 10^−6 ^K^−1^
Thermal Conductivity at 300 K, κ	35.0 Wm^−1^K^−1^
Compressive Strength, σ_+_	1940 + 103 MPa
Compressive Strength, σ_//_	1418 + 281 MPa

Physical, thermal, and mechanical properties of hot-pressed MoAlB in this work.
