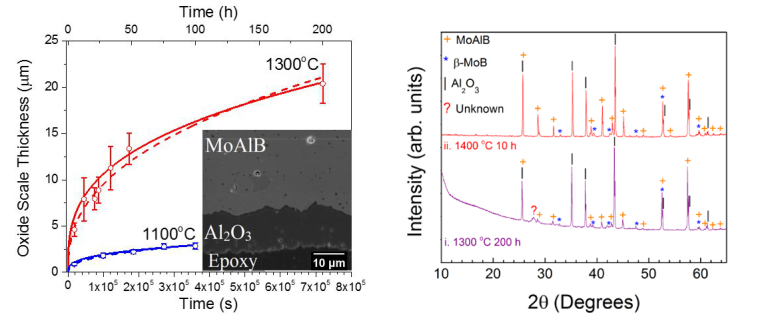# Corrigendum: Synthesis and Characterization of an Alumina Forming Nanolaminated Boride: MoAlB

**DOI:** 10.1038/srep30339

**Published:** 2016-08-19

**Authors:** Sankalp Kota, Eugenio Zapata-Solvas, Alexander Ly, Jun Lu, Omar Elkassabany, Amanda Huon, William E. Lee, Lars Hultman, Steve J. May, Michel W. Barsoum

Scientific Reports 6: Article number: 2647510.1038/srep26475; published online: 05 25 2016; updated: 08 19 2016.

In this Article, the authors ran an XRD experiment on the oxidised samples indicating that the alumina layers formed at temperatures as high as 1300 °C were mostly amorphous and an alumina signal was not detected, as shown in Figure 7b. Since then, new data has suggested that this is incorrect; there is a strong signal for alumina confirming that the oxidized samples were crystalline. The correct Figure 7 appears below as Figure 1. This does not affect the conclusions reached in this Article. As a result,

In the Abstract,

“Unique among the transition metal borides, MoAlB forms a dense, mostly amorphous, alumina scale when heated in air.”

now reads:

“Unique among the transition metal borides, MoAlB forms a dense, alumina scale when heated in air.”

In the Results and Discussion section under subheading ‘Oxidation Resistance’,

“XRD of the samples oxidized for 1300 °C for 200 h (Fig. 7bi) shows faint diffraction peaks corresponding to the formation of β−ΜοΒ and Al_2_O_3_(ICSD #01-071-1125). Clear XRD evidence for the formation of Al_2_O_3_, however, was only obtained when a sample was oxidized at 1400 °C for 10 h (Fig. 7bii). It follows that the alumina layers formed at temperatures as high as 1300 °C were mostly amorphous and quite resistant to crystallization. For example, when Ti_2_AlC, another alumina former, is oxidized, clear and sharp alumina peaks are observed in XRD diffraction patterns of samples oxidized in air at 1000 °C for 120 h^30^. This resistance to crystallization is quite unusual and warrants further work. Notably, when understood, it may be possible to synthesize an amorphous alumina that may flow like glass, an exciting prospect.”

now reads:

“XRD of the samples oxidized for 1300 °C for 200 h (Fig. 7bi) shows diffraction peaks corresponding to the formation of β−ΜοΒ and Al_2_O_3_(ICSD #01-071-1125). The same is true for a sample oxidized at 1400 °C for 10 h (Fig. 7bii). When Ti_2_AlC, another alumina former, is oxidized, clear and sharp alumina peaks are also observed in XRD diffraction patterns of samples oxidized in air at 1000 °C for 120 h^30^.”

In the same section,

“However, more work needs to be done to clearly understand the role of Mo and B during high-temperature oxidation, and their role, if any, in preventing the crystallization of the alumina layer.”

now reads:

“However, more work needs to be carried out to clearly understand the role of Mo and B during high-temperature oxidation.”

In the same section,

“Since cubic oxidation kinetics are rationalized on the basis of crystal growth, it is not clear why the kinetics observed here are cubic. TEM studies are ongoing to understand the exact oxidation mechanism.”

now reads:

“Since cubic oxidation kinetics are rationalized on the basis of crystal growth, it is not surprising that the kinetics observed here are cubic. Nevertheless, TEM studies are ongoing to understand the exact oxidation mechanism.”

These errors have now been corrected in the PDF and HTML versions of the Article.

## Figures and Tables

**Figure 1:**