# Supplementary material for: Synthesis and Characterization of an Alumina Forming Nanolaminated Boride: MoAlB
Source: Sci Rep. 2016 May 25;6:26475. doi: 10.1038/srep26475 (PMC4879536; doi:10.1038/srep26475)
Supplement: Supplementary Information [file srep26475-s1.pdf]

Supplementary Information

Synthesis and Characterization of an Alumina Forming Nanolaminated Boride: MoAlB

Sankalp Kota [1], Eugenio Zapata-Solvas [2], Alexander Ly [3], Jun Lu [4], Omar Elkassabany [1], Amanda Huon [1], William E. Lee [2], Lars Hultman [4], Steve J. May [1], Michel W. Barsoum [1, *]

1 Drexel University, Department of Materials Science & Engineering, Philadelphia, Pennsylvania 19104, United States
2 Centre for Nuclear Engineering, Department of Materials, Imperial College London, London, SW7 2AZ, United Kingdom
3 Drexel University, Department of Mechanical Engineering and Mechanics, Philadelphia, 19104, United States
4 Linköping University, Department of Physics, Chemistry and Biology (IFM), Linköping, SE-58183, Sweden
* Corresponding author: barsoumw@drexel.edu

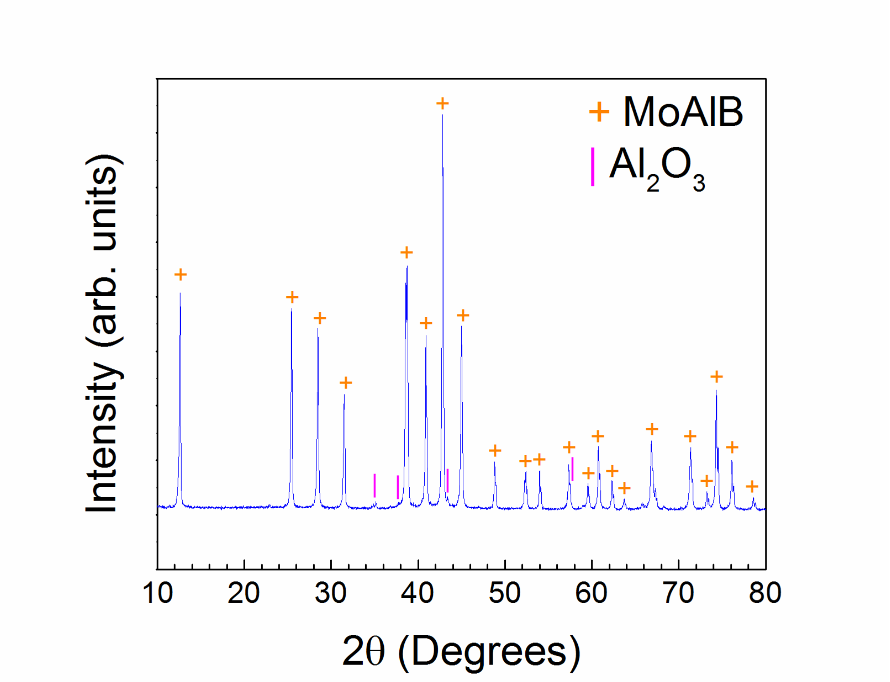

(a)

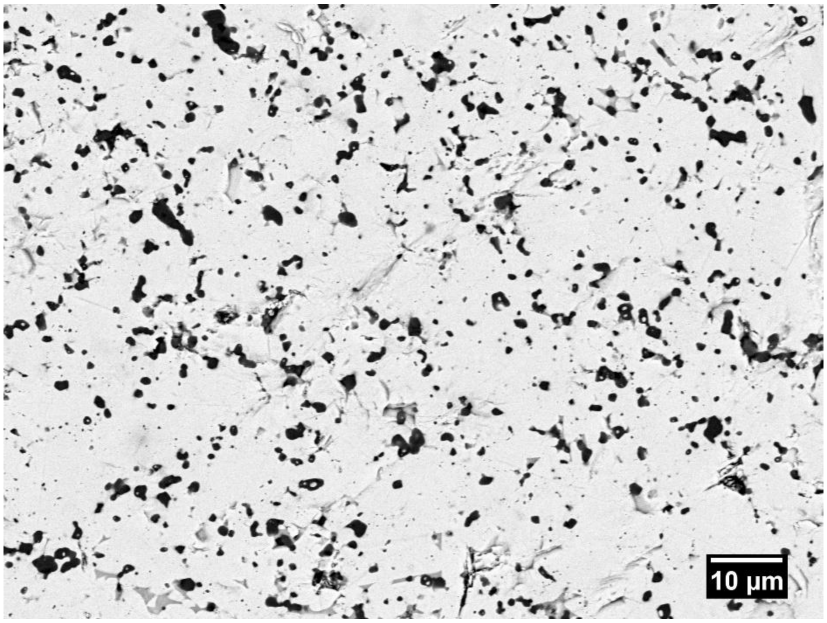

(b)

Fig. S1. (a) XRD pattern of the top surface of the HP2 sample and (b) electron backscatter image of the polished surface of HP2

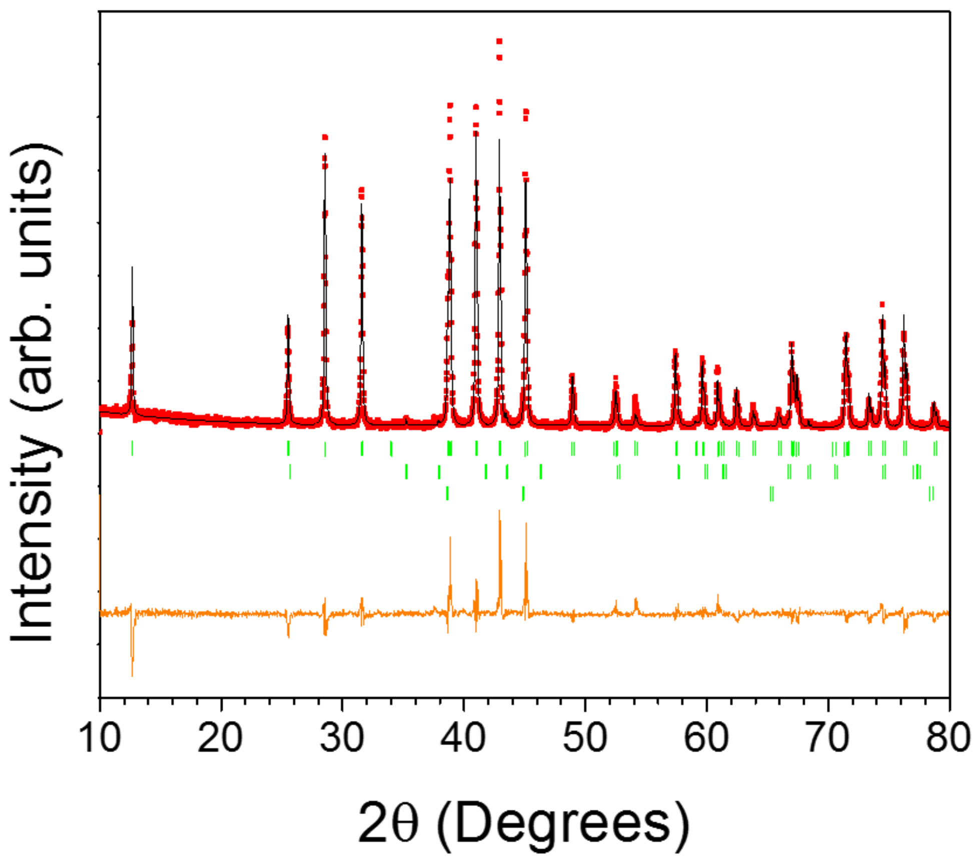

Refinement Details	
χ^2	3.85
**Parameters**	21
**Phases Refined**	3
**Impurities (vol. %)**	Al_2O_3 (8 %)<br>Al (2 %)

Fig. S2. Rietveld refinement of MoAlB powders synthesized in a tube furnace with the observed pattern (red) and calculated pattern (black), and the difference between observed and calculated intensities (orange). Vertical green dashes show calculated diffraction positions of MoAlB (top row), Al_2O_3 (middle row), and Al (bottom row).

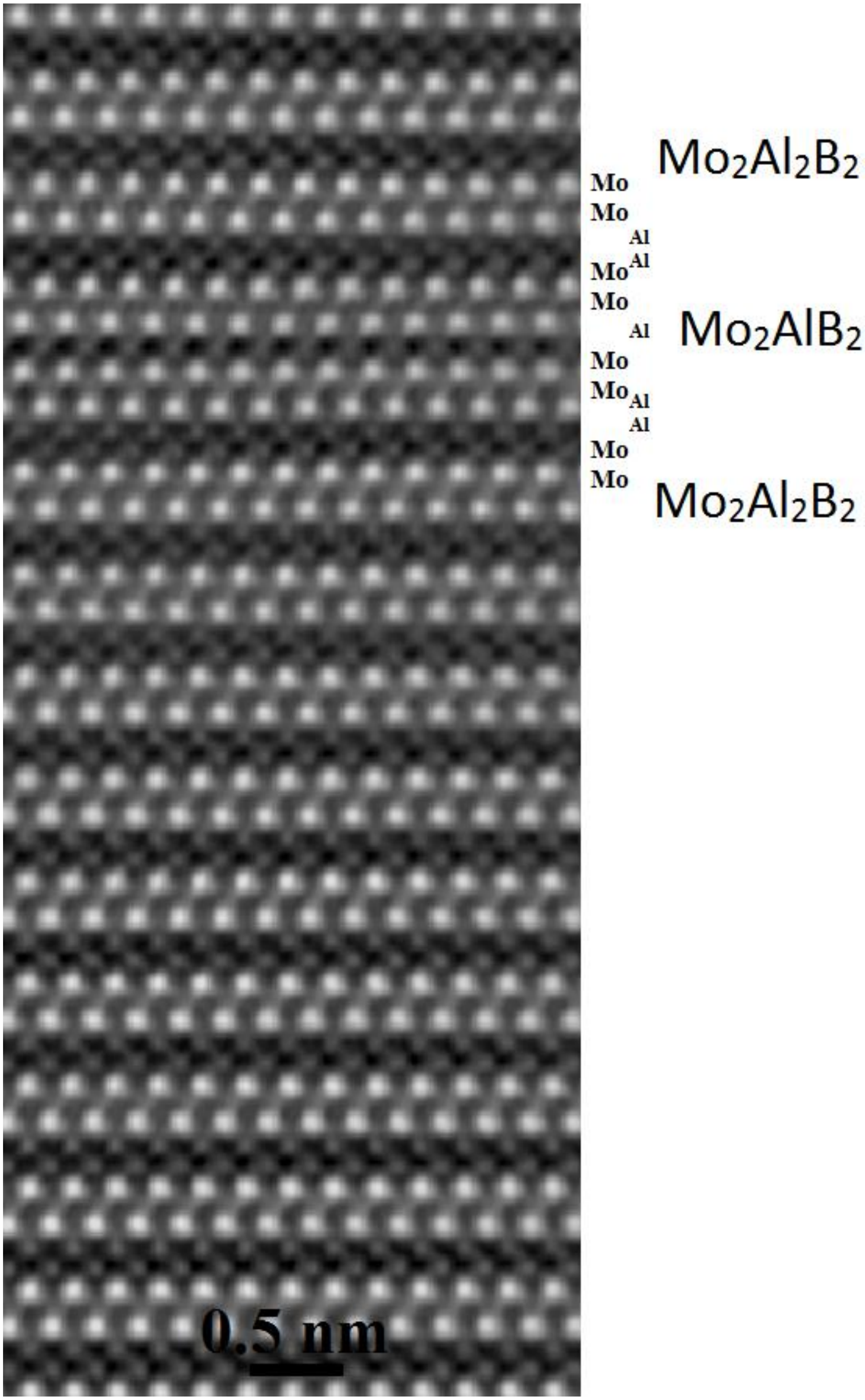

Fig. S3. HRSTEM image of a stacking fault viewed along the [100] zone axis where only a single Al layer separates a Mo-B block instead of the expected two Al layers.

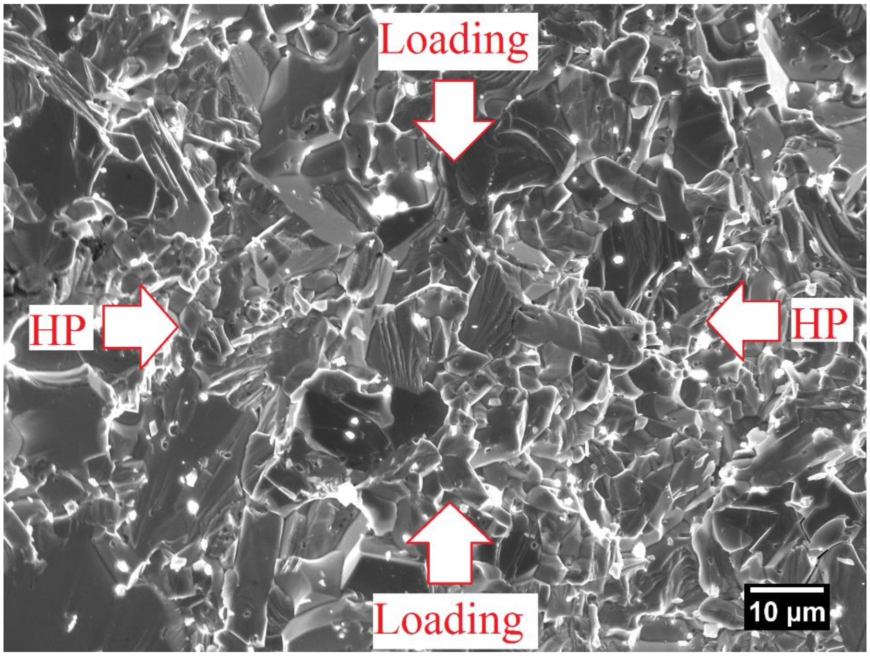

(a)

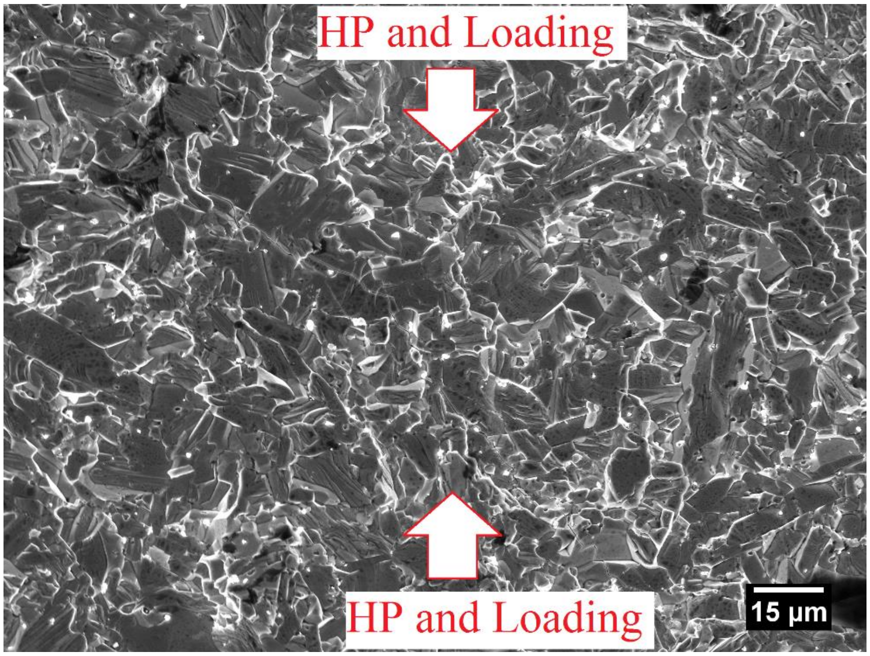

(b)

Fig. S4. Fracture surface from compression testing when sample is loaded (a) perpendicular to hot pressing direction and (b) parallel to hot pressing direction. ‘HP’ in the images indicates the direction the samples were hot-pressed.

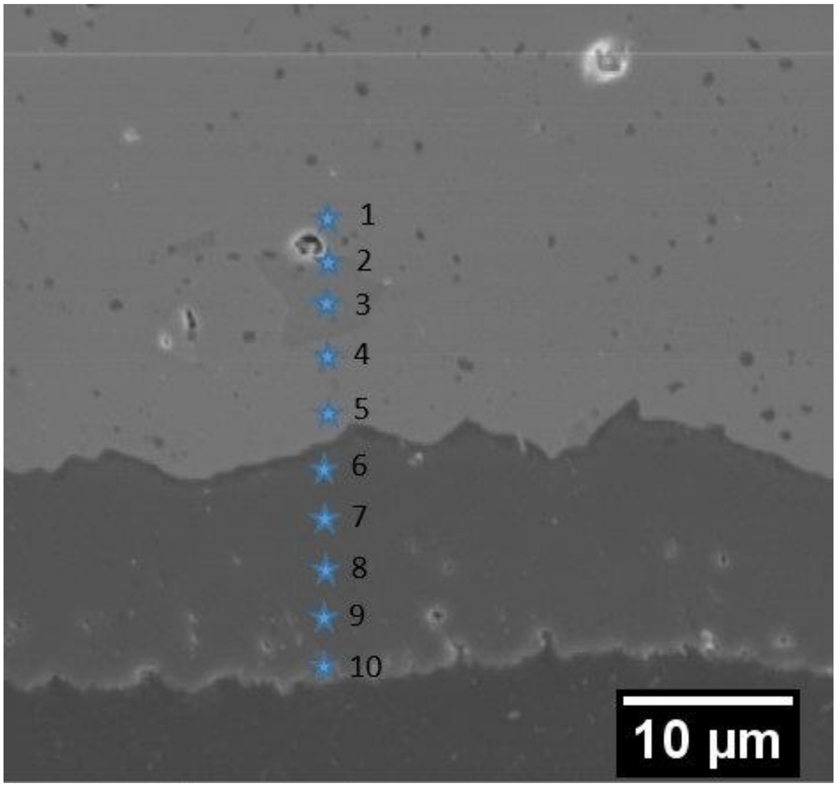

Point	Mo (at %)	Al (at %)	O (at %)
1	49.75	50.25	-
2	37.79	62.21	-
3	25.94	74.06	-
4	49.47	50.53	-
5	49.74	50.26	-
6	-	37.61	62.39
7	-	38.19	61.81
8	-	36.57	63.43
9	-	36.39	63.61
10	-	36.27	63.73

Fig. S5. SEM micrograph of the cross-section of MoAlB oxidized after 200 h at 1300 ^oC. The blue stars correspond to the points where EDS point scans were performed. b) Atomic content of Mo, Al, and O on the cross-section of a bulk MoAlB sample oxidized at 1300 ^oC. The points in table correspond to the points shown in a.

Sample	a (Å)	b (Å)	c (Å)	Reference
HP4 cross-section	3.213	13.978	3.103	This work
MoAlB Powders	3.208	13.961	3.100	This work
Okada *et al.*	3.213	13.986	3.103	Ref. 1
Ade *et al.*	3.199	13.922	3.094	Ref. 2

Table S1. Comparison of lattice constants obtained from Rietveld refinement of XRD patterns of the HP4 cross-section and MoAlB powders made in the tube furnace with those reported by Okada [1] and Ade *et al.*[2]

Additional References

1. Okada, S. Synthesis, Crystal Structure and Characterizations of the Ternary Borides TMAlB (TM=Mo,W) with UBC Type Structure. *Trans. Kokushikan Univ. Fac. Eng.* 7–12 (1998).

2. Ade, M. & Hillebrecht, H. Ternary Borides Cr_2AlB_2 , Cr_3AlB_4 , and Cr_4AlB_6: The First Members of the Series $(CrB_2)_nCrAl$ with n = 1, 2, 3 and a Unifying Concept for Ternary Borides as MAB-Phases. *Inorg. Chem.* **54,** 6122–6135 (2015).
